# The Effect of Chronic Rhinosinusitis on the Staging and Prognosis of Extranodal Natural Killer/T-Cell Lymphoma: A Single-Center Retrospective Analysis

**DOI:** 10.3389/fonc.2022.878559

**Published:** 2022-04-05

**Authors:** Tingting Lei, Yu Chang, Lei Zhang, Mingzhi Zhang

**Affiliations:** Department of Oncology, The First Affiliated Hospital of Zhengzhou University; Lymphoma Diagnosis and Treatment Center of Henan Province, Zhengzhou, China

**Keywords:** extranodal natural killer/T-cell lymphoma (ENKTL), chronic rhinosinusitis (CRS), stage, prognosis, effect

## Abstract

Clinically, extranodal natural killer/T-cell lymphoma (ENKTL) patients frequently had a history of chronic rhinosinusitis (CRS) before onset, and the correlation between the two diseases has not been systematically reported at present. In this study, we applied the method—retrospective analysis—to explore the relationship between CRS and ENKTL. We collected clinical data and the length of CRS history before onset in 214 patients diagnosed with ENKTL and found that the length of CRS history was correlated with the stage of 182 ENKTL patients whose primary sites were upper aerodigestive tract (UAT) (*χ*^2^ = 21.317, *p* = 0.046, *n* = 182); the Spearman correlation coefficient was 0.162 (*p* = 0.029). There was no significant difference in stage of the non-UAT-ENKTL patients (*χ*^2^ = 18.910, *p* = 0.091, *n* = 32). The COX multivariate regression analysis showed that CRS history was an independent prognostic predictor for PFS of the UAT-ENKTL patients (*p* = 0.004), and patients without CRS had significantly better PFS than the more than 15 years CRS history group (*p* = 0.001). Our findings suggested that we should not ignore the existence of chronic inflammation of the nasal cavity in ENKTL patients. It is better to treat CRS as soon as possible in clinical practice to reduce the possibility of the occurrence or progression of UAT-ENKTL.

## Introduction

Extranodal natural killer/T-cell lymphoma (ENKTL) is an unusual subtype of non-Hodgkin’s lymphoma (NHL) with distinctive clinicopathological and aggressive clinical features (1). This disease is more common among Asians and South Americans than in Western populations (2). The Epstein–Barr virus (EBV) infection plays an important role in the incidence and progression of ENKTL (3, 4). Most cases of ENKTL occurs in the upper aerodigestive tract (UAT); typically presents in the nasal cavity or nasopharynx with a common symptom of nasal obstruction, epistaxis, or nasal-space-occupying lesions; and progressively invades adjacent soft tissues and bone as the disease progresses (3, 5, 6). Other cases that present outside the UAT have similar histological features; their common primary sites include the skin, testis, gastrointestinal tract, and so on (6, 7). In recent years, despite the L-asparaginase (L-Asp)-based chemotherapy, radiotherapy, or immunotherapy brought a better response rate, due to the highly aggressive features of ENKTL, the prognosis of patients was still poor (1, 4, 8).

Chronic rhinosinusitis (CRS) is a common disease that presents as persistent inflammation of the mucosa of the nose and paranasal sinuses (9, 10). Clinically, we observed that ENKTL patients whose primary site was the UAT frequently had a history of CRS before onset. Previous studies have demonstrated that chronic inflammation is intimately correlated with the incidence and progression of various tumors (11–13). However, there have been no studies specifically focused on the correlation between ENKTL and CRS. In this study, we aimed to elucidate the relationship between CRS and the clinical characteristics, response rates, and survival outcomes for ENKTL.

## Patients and Methods

### Patients

All 214 patients diagnosed with ENKTL (nasal type) at the Lymphoma Diagnosis and Treatment Center of Henan Province, the First Affiliated Hospital of Zhengzhou University between May 2008 to March 2021 were systematically reviewed in this study. The inclusion criteria were as follows: (a) diagnosis of ENKTL (nasal type) according to histological features and immunophenotypic characteristics as described in the WHO Classification of Tumors of Hematopoietic and Lymphoid Tissues (14); (b) a set of complete clinical materials, laboratory, imaging, and treatment data; and (c) no concomitant other types of malignancies. This study was approved by the Ethics Committees of the First Affiliated Hospital of Zhengzhou University; the ethics number is 2021-KY-0590.

The clinical characteristics of all 214 patients were collected, including age, sex, the length of the history of CRS before ENKTL onset, the Chinese Southwest Oncology Group (CSWOG) and Asia Lymphoma Study Group (ALSG) ENKTL system (CA) stage (15), B symptoms, nomogram-revised index (NRI) score, prognostic index for natural killer/T-cell lymphoma-EBV (PINK-E) score, EBV-DNA level, lactate dehydrogenase (LDH) level, β_2_-microglobulin (β_2_-MG) level, bone marrow involvement, treatment modalities, date of initial treatment, response to therapy, date of relapse/progression, date of the last follow-up, and survival status. According to the European Guidelines for the Diagnosis and treatment of sinusitis nasal polyps (EPOS 2020), the CRS is diagnosed by existing symptoms of nasal obstruction, rhinorrhea, epistaxis, or hyposmia, and lasts 12 weeks at least. The results of nasal endoscopy, sinus computed tomography (CT), or magnetic resonance imaging (MRI) were as references (16). The ENKTL patient who excludes a CRS diagnosis must satisfy at least one of the following two conditions in addition to denying any persistent CRS-related symptoms: (a) medical records showed no symptoms of CRS or the onset of related symptoms for <12 weeks, and (b) imaging data before initial treatment showed no signs of CRS.

### Treatment Modalities

The initial treatment modalities of all 214 patients included chemotherapy alone, radiotherapy alone, combined radiotherapy, and chemotherapy. According to the CA staging system and NCCN Clinical Practice Guidelines in Oncology, patients in stages I–II were mostly treated with the combination of radiotherapy and chemotherapy, and radiotherapy alone was feasible for some patients. Patients in stages III–IV were mostly treated with chemotherapy alone (17). The median radiation dose was 50 Gy (range, 22–60 Gy). The main chemotherapy protocols were DDGP protocol (Dexamethasone, Cisplatin, Gemcitabine, and Pegaspargase), SMILE protocol (Dexamethasone, Methotrexate, Ifosfamide, Pegaspargase, and Etoposide), and P-GEMOX protocol (Pegaspargase, Gemcitabine, and Oxaliplatin). The detailed information is specified in **Table 1**.

**Table 1:** Initial treatment modalities based on the subtype and CA staging.

Treatment modalities	UAT-ENKTL, *n* (%)		Non-UAT-ENKTL, *n* (%)
**Stage Ⅰ/Ⅱ**	n = 117		n = 11
**Chemotherapy alone**			
DDGP	21		6
SMILE	1		0
VIPD	2		0
ELSE	1		0
**Combined radiotherapy and chemotherapy^a^**			
DDGP	64	4	
VIPD	12	0	
DICEL	2	0	
CHOP-like	2	0	
AspaMetDex	1	1	
**Radiotherapy alone**	11	0	
**Stage III/IV**	n = 65	n = 21	
**Chemotherapy alone**			
DDGP	53	16	
SMILE	5	2	
P-GEMOX	1	3	
VIPD	1	0	
DICEL	1	0	
CHOP-like	4	0	

a Combined treatment of three to six cycles of chemotherapy in conjunction with radiotherapy.

DDGP, Dexamethasone, Cisplatin, Gemcitabine, Pegaspargase; SMILE, Dexamethasone, Methotrexate, Ifosfamide, Pegaspargase, Etoposide; P-GEMOX, Pegaspargase, Gemcitabine, Oxaliplatin; VIPD, Etoposide, Cisplatin, Ifosfamide, Dexamethasone; DICEL, Dexamethasone, Ifosfamide, Cisplatin, Etoposide, L-asparaginase; CHOP-like, Cyclophosphamide, Doxorubicin, Vincristine, Dexamethasone, Etoposide/L-asparaginase; AspaMetDex, L-asparaginase, Methotrexate, Dexamethasone; UAT, upper aerodigestive tract; ENKTL, natural killer/T-cell lymphoma, nasal type.

### Evaluation

The response evaluation was done every two cycles. Methods of assessing efficacy included CT or positron emission tomography-computed tomography (PET-CT); if necessary, we combined with color Doppler ultrasound, MRI, and bone marrow puncture. Based on the Lugano Classification, the clinical response of patients was categorized as complete response (CR), partial response (PR), stable disease (SD), or progressive disease (PD). The objective response rate (ORR) was defined as the percentage of patients who achieved CR or PR (18). The long-term clinical efficacy was assessed with overall survival (OS) and progression-free survival (PFS). The disease control rate (DCR) was calculated from the percentage of CR + PR+ SD patients among all individuals. OS was defined as the time interval starting from the day of therapy to the final follow-up or death. PFS was calculated from the date of starting therapy to the date of disease progression or final follow-up.

### Statistical Analyses

All statistical analyses were performed using IBM SPSS 26.0 software and GraphPad Prism 8 software. The difference in categorical variables was compared by Pearson chi-square analysis. Kaplan–Meier curves and the log-rank test were used to evaluate OS and PFS. Prognostic risk factors were estimated with univariate analysis. Significant factors were further estimated by multiple factors analysis of the Cox proportional hazards regression model. A level of *p* < 0.05 was considered statistically significant, and all tests were two-sided.

## Results

### Patient Characteristics

The characteristics of all 214 patients are listed in **Table 2**. The median age was 43 years (range, 11–76 years), and the ratio of male to female was 2.29:1. Eighty-five percent of the patients (*n* = 182) were diagnosed as UAT-ENKTL. B symptoms were presented in 89 patients (41.6%). Fifty patients (23.4%) had stage IV disease. Elevated EBV-DNA level was detected in 60 patients (38.0%) at first diagnosis. 107 patients had an NRI score of 3–5. In 168 patients (78.5%), a history of CRS existed before being diagnosed as UAT-ENKTL.

**Table 2:** Characteristics of 214 ENKTL patients.

Characteristics	*n* (%)	Characteristics	*n* (%)
**Sex**		**CA stage**	
Male	149 (69.6)	I	58 (27.1)
Female	65 (30.4)	II	70 (32.7)
**Age (years)**		III	36 (16.8)
≤60	182 (85.0)	IV	50 (23.4)
>60	32(15.0)	**LDH level**	
**B symptoms**		Normal	131(61.2)
Yes	89 (41.6)	Elevated	83 (38.8)
No	125 (58.4)	**β_2_-MG level**	
**NRI score**		Normal	154(72.0)
0–2	107(50.0)	Elevated	60 (28.0)
3–5	107(50.0)	**Primary sites of tumor**	
**PINK-E score**		UAT	182(85.0)
0–1	157(73.4)	Non-UAT	32 (15.0)
2–3	57 (26.6)	**Treatment modalities**	
**EBV-DNA level**		Radiotherapy	11 (5.1)
Normal	154(72.0)	Chemotherapy	117 (54.7)
Elevated	60 (28.0)	Radiotherapy and chemotherapy	86 (40.2)
**Bone marrow involvement**		**CRS history**	
Absence	205(95.8)	Yes	168(78.5)
Presence	9 (4.2)	No	46(21.5)

ENKTL, natural killer/T-cell lymphoma, nasal type; CA stage, the Chinese Southwest Oncology Group (CSWOG) and Asia Lymphoma Study Group (ALSG) ENKTL system (CA) stage; IPI, International Prognostic Index; PINK-E, the prognostic index for natural killer/T-cell lymphoma-EBV; NRI, nomogram-revised index; EBV, Epstein–Barr virus; LDH, lactate dehydrogenase; β_2_-MG, β2-microglobulin; UAT, upper aerodigestive tract; CRS, chronic rhinosinusitis.

For the ENKTL patients whose primary site was UAT, 182 patients were divided into five groups based on the length of their history of CRS before onset obtained from follow-up. As was shown in **Table 3**, the five groups were named as non-CRS group, 0–5 years group, 5–10 years group, 10–15 years group, and more than 15 years group. We found that there was no obvious difference between the most clinical characteristics (*p* > 0.05) except for the CA stage (*χ*^2^ = 21.317, *p* = 0.046) in the five groups; the Spearman correlation coefficient was 0.162 (*p* = 0.029). There was no significant difference in the CA stage of the non-UAT-ENKTL patients (*χ*^2^ = 18.910, *p* = 0.091).

**Table 3:** Comparison of clinical characteristics of 182 UAT-ENKTL patients with non-CRS group, CRS history of 0–5 years group, 5–10 years group, 10–15 years group, and more than 15 years group.

Characteristics	non-CRS group, *n* (%)	CRS history				χ^2^	p-value
		0–5 years group, *n* (%)	5–10 years group, *n* (%)	10–15 years group, *n* (%)	More than 15 years group, *n* (%)		
**Sex**						6.619	0.157
Male	21(70.0)	51(71.8)	22(71.0)	12(52.2)	23(85.3)		
Female	9(30.0)	20(28.2)	9(29.0)	11(47.8)	4(14.8)		
**Age (years)**						2.632	0.621
≤60	23(76.7)	60(84.5)	28(90.3)	18(78.3)	23(85.2)		
>60	7(23.3)	11(15.5)	3(9.7)	5(21.7)	4(14.8)		
**CA stage**						21.317	0.046^*^
I	14(46.7)	22(28.2)	4(12.9)	6(26.1)	6(22.2)		
II	6(20.0)	32(45.1)	12(38.7)	12(52.2)	6(22.2)		
III	5(16.7)	8(11.3)	7(22.6)	3(13.0)	8(29.6)		
IV	5(16.7)	11(15.5)	8(25.8)	2(8.7)	7(25.9)		
**B symptoms**						3.671	0.452
Yes	8(26.7)	31(43.7)	3(41.9)	8(34.8)	13(48.1)		
No	22(73.3)	40(56.3)	18(58.1)	15(65.2)	14(51.9)		
**NRI score**						4.349	0.361
0–2	16(53.3)	36(50.7)	17(54.8)	16(69.6)	11(40.7)		
3–5	14(46.7)	35(49.3)	14(45.2)	7(30.4)	16(59.3)		
**PINK-E score**						7.745	0.101
0–1	25(83.3)	58(81.7)	20(64.5)	21(91.3)	19(70.4)		
2–3	5(16.7)	13(18.3)	11(35.5)	2(8.7)	8(29.6)		
**EBV-DNA level**						2.514	0.642
Normal	24(80.0)	50(70.4)	21(67.7)	19(82.6)	20(74.1)		
Elevated	6(20.0)	21(29.6)	10(32.3)	4(17.4)	7(25.9)		
**LDH level**						1.381	0.847
Normal	21(70.0)	44(62.0)	18(58.1)	16(69.6)	17(63.0)		
Elevated	9(30.0)	27(38.0)	13(41.9)	7(30.4)	10(37.0)		
**β_2_-MG level**						2.311	0.679
Normal	20(66.7)	57(80.3)	24(77.4)	18(78.3)	20(74.1)		
Elevated	10(33.3)	14(19.7)	7(22.6)	5(21.7)	7(25.9)		
**Bone marrow involvement**						0.146	0.997
Presence	1(3.3)	2(2.8)	1(3.2)	1(4.3)	1(3.7)		
Absence	29(96.7)	69(97.2)	30(96.8)	22(95.7)	26(96.3)		
**Treatment modalities**						7.191	0.516
Radiotherapy	4(13.3)	3(4.2)	2(6.5)	3(13.6)	0(0.0)		
Chemotherapy	11(36.7)	30(42.3)	15(48.4)	8(36.4)	12(44.4)		
Radiotherapy and	15(50.0)	8(53.5)	14(45.2)	12(50.0)	15(55.6)		
chemotherapy							

ENKTL, natural killer/T-cell lymphoma, nasal type; UAT, upper aerodigestive tract; CRS, Chronic rhinosinusitis; CA stage, the Chinese Southwest Oncology Group (CSWOG) and Asia Lymphoma Study Group (ALSG) ENKTL system (CA) stage; PINK-E, the prognostic index for natural killer/T-cell lymphoma-EBV; NRI, nomogram-revised index; EBV, Epstein–Barr virus; LDH, lactate dehydrogenase; β_2_-MG, β2-microglobulin.

^*^p < 0.05.

### Response

All 182 patients of UAT-ENKTL accepted initial treatments including chemotherapy alone (*n* = 76), radiotherapy alone (*n* = 12), and combined radiotherapy and chemotherapy (*n* = 94). The overall response rate (ORR) was 76.9%, with complete response (CR) achieved by 102 patients (56.0%) and partial response (PR) achieved by 38 patients (20.9%). The disease control rate (DCR) was 82.4%, with stable disease (SD) of 5.5% (*n*=10). There was no obvious statistical difference in response rates of initial treatment between the non-CRS group, 0–5 years group, 5–10 years group, 10–15 years group, and more than 15 years group (ORR, 70.0% vs. 74.6% vs. 80.6% vs. 78.3% vs. 85.2%, *p* = 0.677; DCR, 80.0% vs. 78.9% vs. 87.1% vs. 87.0% vs. 85.2%, *p* = 0.795; CR, 53.3% vs. 57.7% vs. 54.8% vs. 47.8% vs. 63.0%, *p* = 0.853).

### Survival Outcome and Prognosis

**Figure 1:**
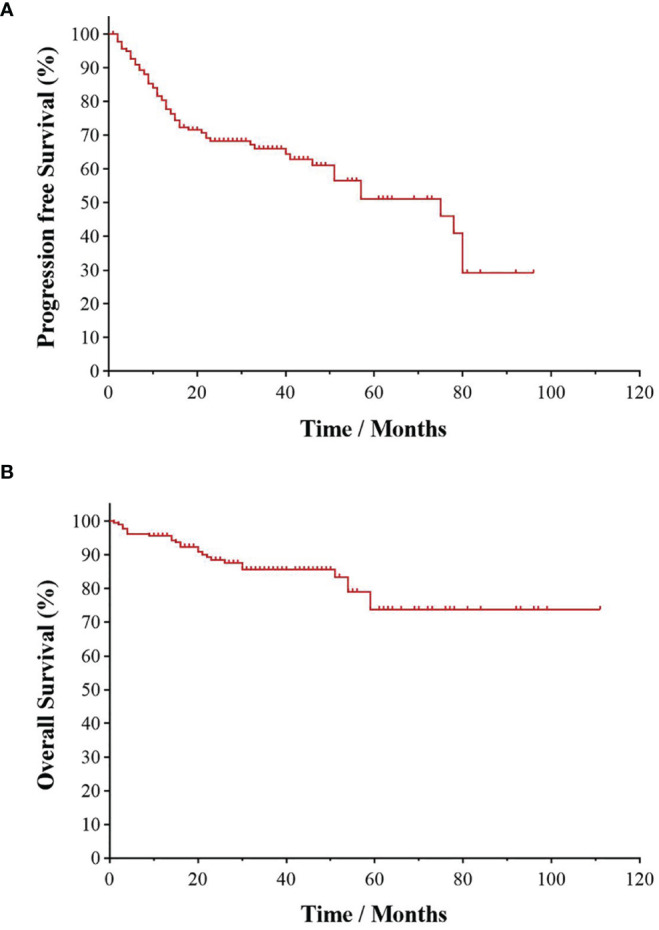
Kaplan–Meier survival curve of UAT-ENKTL patients. Overall survival **(A)** and progression-free survival **(B)** in the total 182 of UAT-ENKTL patients. ENKTL, natural killer/T-cell lymphoma, nasal type; UAT, upper aerodigestive tract.

**Figure 2:**
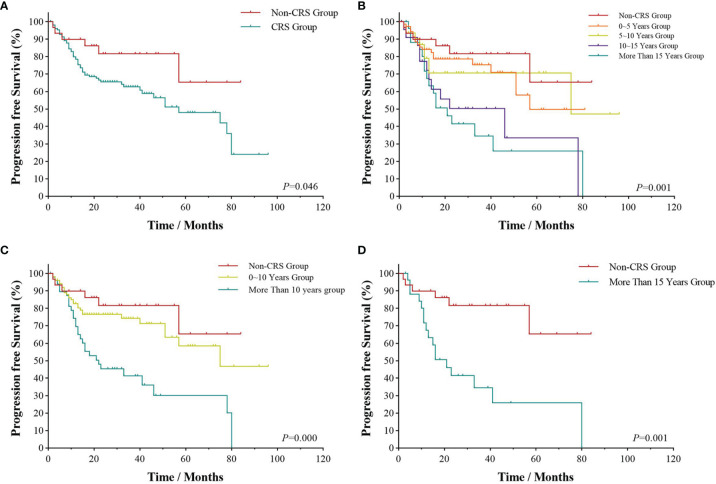
Survival analyses by CRS history. Progression-free survival **(A)** of UAT-ENKTL patients with non-CRS group versus CRS group; progression-free survival **(B)** of UAT-ENKTL patients according to the length of CRS history (non-CRS group, 0–5 years group, 5–10 years group, 10–15 years group, more than 15 years group); progression-free survival **(C)** of non-CRS group, 0–10 years group, and more than 10 years group of all 182 UAT-ENKTL patients; progression-free survival **(D)** of UAT-ENKTL patients with non-CRS group and more than 15 years group. CRS, chronic rhinosinusitis; ENKTL, natural killer/T-cell lymphoma, nasal type; UAT, upper aerodigestive tract.

**Figure 3:**
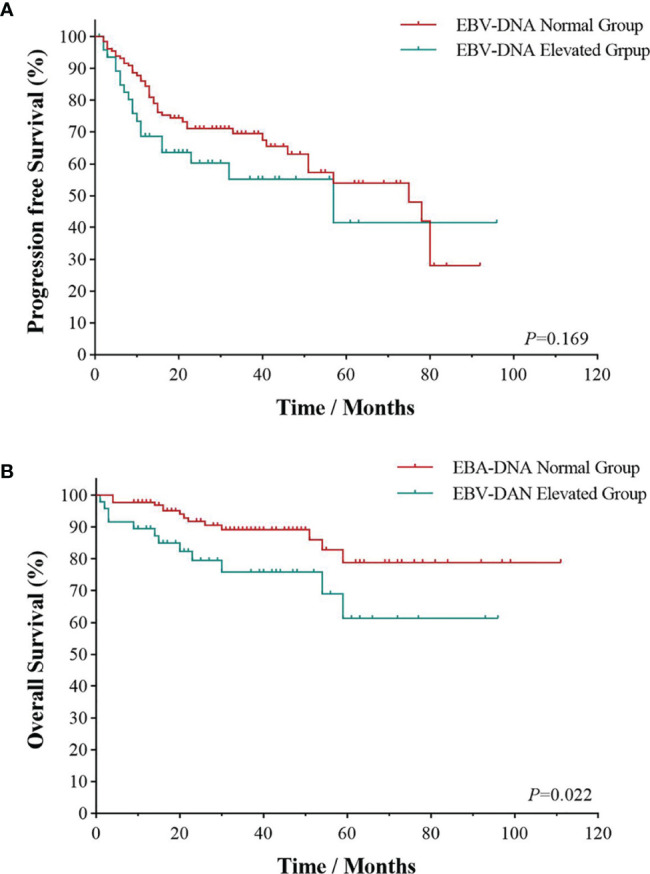
Survival analyses by EBV-DNA. Progression-free survival **(A)** and overall survival **(B)** of EBV-DNA level of UAT-ENKTL patients. ENKTL, natural killer/T-cell lymphoma, nasal type; UAT, upper aerodigestive tract; EBV, Epstein–Barr virus.

All patients were followed up to January 1, 2022, with a median follow-up time of 28.5 months. As shown in **Figure 1**, the 3- and 5-year PFS were 66.0% and 51.1%, and 3- and 5-year OS were 85.1% and 73.4%, respectively, as illustrated in **Figures 1A, B**. The significant factors of PFS or OS with univariate survival analysis are listed in **Table 4**. The 5-year PFS of UAT-NKTCL patients in the non-CRS and CRS groups were 65.3% and 47.9%, respectively (*χ*^2^ = 21.317, *p* = 0.046), as illustrated in **Figures 2A**. No significant difference was noted in OS between the two groups (*χ*^2^ = 0.025, *p* = 0.874), and significant differences were noted in PFS between the groups of UAT-ENKTL patients according to the length of CRS history (*p* = 0.001; *p* < 0.001), as illustrated in **Figures 2B, C**. Moreover, the UAT-ENKTL patients with the non-CRS group have better PFS than the more than 15 years CRS history group, which were significantly different (*p* = 0.001), as illustrated in **Figure 2D**. The OS was significantly higher in the EBV-DNA normal group than EBV-DNA elevated group (*χ^2^* = 5.244, *p* = 0.022). However, no survival difference was found in PFS between the two groups (*p* = 0.169), as illustrated in **Figures 3A, B**.

**Table 4:** Univariate analysis of prognostic factors for PFS and OS.

Prognostic factors	PFS		OS	
	*HR (95% CI)*	*p-*value	*HR (95% CI)*	*p*-value
Sex	1.075 (0.634–1.824)	0.787	0.992 (0.431–2.285)	0.985
Age	1.505 (0.766–2.956)	0.297	2.458 (0.785–7.701)	0.034^*^
CRS history	2.273 (1.218–4.243)	0.046^*^	1.082 (0.398–2.940)	0.874
CA stage	2.004 (1.178–3.412)	0.004^*^	4.115 (1.798–9.415)	0.000^*^
B symptoms	1.128 (0.686–1.858)	0.627	1.787 (0.822–3.881)	0.135
NRI score	1.399 (0.853–2.294)	0.171	4.590(2.107–10.001)	0.000^*^
PINK-E score	1.170 (0.604–2.264)	0.617	4.249 (1.519–11.890)	0.000^*^
EBV-DNA level	1.446 (0.805–2.598)	0.169	2.383 (0.997–5.697)	0.022^*^
LDH level	1.597 (0.929–2.746)	0.078	2.222 (0.968–5.100)	0.035^*^
β_2_-MG level	1.307 (0.721–2.368)	0.337	2.855 (1.147–7.108)	0.005^*^
Bone marrow involvement	1.325 (0.716–2.453)	0.842	0.332 (0.011–9.680)	0.256
Treatment modalities	–	0.054	–	0.000^*^

CI, confidence interval; HR, hazard ratio; CRS, Chronic rhinosinusitis; CA stage, the Chinese Southwest Oncology Group (CSWOG) and Asia Lymphoma Study Group (ALSG) ENKTL system (CA) stage; PINK-E, the prognostic index for natural killer/T cell lymphoma-EBV; NRI, nomogram-revised index; EBV, Epstein-Barr virus; LDH, lactate dehydrogenase; β_2_-MG, β_2_-microglobulin; PFS, progression-free survival; OS, overall survival.

^*^p < 0.05.

**Figure 4:**
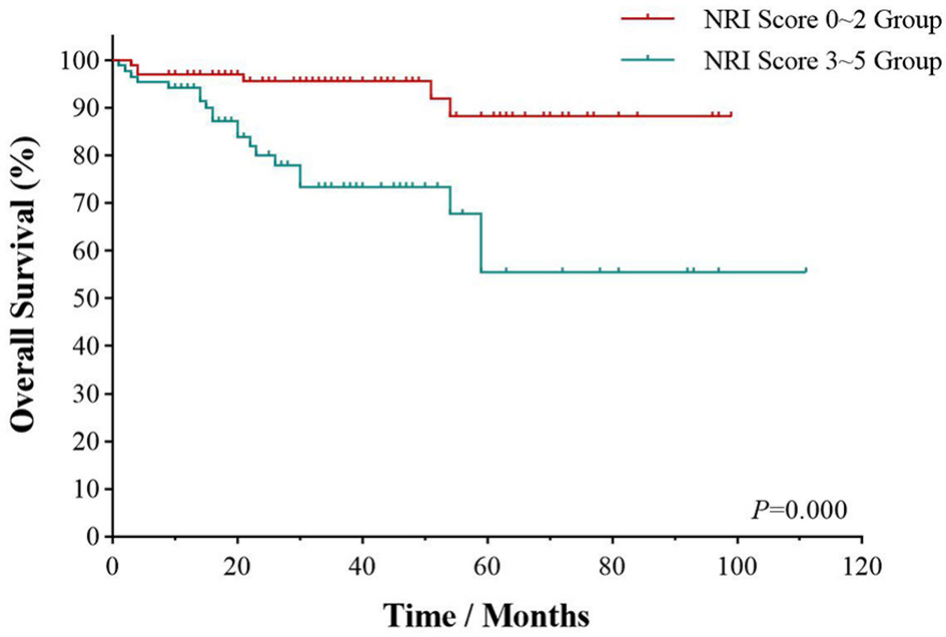
Survival analyses by NRI score. Overall survival of UAT-ENKTL patients according to NRI score (0–2 group versus 3–5 group). ENKTL, natural killer/T-cell lymphoma, nasal type; UAT, upper aerodigestive tract; NRI, nomogram-revised index.

The multivariate survival analysis validated by the 10-fold cross method indicated that CA stage (*HR* = 1.920; *95% CI*, 1.132–3.258, *p* = 0.004) and CRS history (*p* = 0.004) was an independently prognostic predictor for PFS in **Table 5**. NRI score was an independently prognostic predictor for OS (*HR* = 4.060; *95% CI*, 1.821–9.050, *p* = 0.001) (**Figure 4**).

**Table 5:** Multivariate analysis of prognostic factors for PFS and OS.

Prognostic factors	PFS		OS	
	*HR (95% CI)*	*p-*value	*HR (95% CI)*	*p-*value
Age	–	–	–	0.466
CA stage	1.920 (1.132–3.258)	0.016^*^	–	0.122
NRI score	–	0.506	4.060 (1.821–9.050)	0.001^*^
PINK-E score	–	0.569	–	0.113
EBV-DNA level	–	0.202	–	0.497
LDH level	–	0.134	–	0.700
β_2_-MG level	–	–	–	0.105
Treatment modalities	–	0.188	–	0.098
CRS history	–	0.004^*^	–	0.772
0–5 years group vs. Non-CRS group	1.723 (0.685–4.337)	0.248	–	0.965
5–10 years group vs. Non-CRS group	1.450 (1.450–4.093)	0.483	–	0.306
10–15 years group vs. Non-CRS group	4.143 (4.143–1.543)	0.005^*^	–	0.820
More than 15 years group vs. Non-CRS group	3.596 (3.596–1.409)	0.007^*^	–	0.297

CI, confidence interval; HR, hazard ratio; CRS, Chronic rhinosinusitis; CA stage, the Chinese Southwest Oncology Group (CSWOG) and Asia Lymphoma Study Group (ALSG) ENKTL system (CA) stage; PINK-E, the prognostic index for natural killer/T cell lymphoma-EBV; NRI, nomogram-revised index; EBV, Epstein–Barr virus; LDH, lactate dehydrogenase; β2-MG, β2-microglobulin; PFS, progression-free survival; OS, overall survival.

^*^p < 0.05.

## Discussion

As an aggressive non-Hodgkin’s lymphoma, ENKTL has a high degree of malignancy and poor prognosis. Early symptoms of ENKTL patients are often atypical, mainly manifested as nasal congestion, nasal bleeding, nasal polyps, etc. (19, 20). It is easy to be misdiagnosed because of the similar symptoms between ENKTL of early stage and CRS. Clinically, we found that ENKTL patients often have a history of CRS before onset, and the correlation between the two diseases has not been systematically reported at present. In this study, the relationship between CRS and UAT-ENKTL was systematically analyzed for the first time, and it was found that the course of CRS was correlated with the CA stage of the UAT-ENKTL patients. CRS history as an independent prognostic predictor for PFS; the UAT-ENKTL patients without CRS has significantly better PFS than the patients with CRS history for more than 15 years, but there was no significant effect on the short-term efficacy and OS of UAT-ENKTL patients.

Chronic inflammation plays an important role in the occurrence and development of tumors. The relationship between chronic inflammation and tumors has been extensively studied since Rudolf Virchow noticed white blood cells in tumor tissues and believed that tumors originated from the site of chronic inflammation in 1863 (21). Many studies have shown that 15%–20% of tumors are related to chronic inflammation (11, 22). Over the past 20 years, a large amount of experimental and clinical evidence has shown that chronic inflammations act on all stages of occurrence and development of tumors. Chronic inflammations drive cell transformation and tumor initiation through gene mutation or epigenetic mechanism and promote tumor cell proliferation, anti-apoptosis, invasion, and metastasis by establishing a tumor microenvironment (TME). In addition, the effective immune response to tumors can be prevented by establishing an immune suppression mechanism (23, 24).

Our results indicated that the presence of CRS affects the clinical characteristics and prognosis of UAT-ENKTL patients to a certain extent, and CRS may be one of the important factors promoting the occurrence and progression of ENKTL. Although there is not enough direct evidence *in vitro* and *in vivo* to prove the correlation between the two, a large amount of indirect evidence has proved that CRS can have systemic effects either directly through the release of inflammatory mediators or indirectly by altering inflammatory homeostasis and the delicate balance of the organism with the commensal flora and promote the formation and progression of ENKTL (12).

First, in UAT-ENKTL, CRS altered local control of tissue homeostasis and genetic stability, inducing DNA damage and genomic instability. In inflammatory tissues, increased tissue damage, repair, and cell proliferation increase the likelihood of mutations or chromosomal translocations during mitosis. Activated inflammatory cells are the source of reactive oxygen species (ROS) and reactive nitrogen intermediates (RNI), which induce DNA mutations and epigenetic modifications of proto-oncogenes and tumor suppressor genes and increase genetic instability and DNA replication error rate (24). Among these, nitric oxide (NO) and its derivatives can regulate the tumor suppressor gene Tp53. Under normal circumstances, NO can stabilize and activate Tp53 and induce apoptosis of cancer cells, and when Tp53 is mutated, NO can help tumor cell proliferation (25). Through analysis of second-generation gene sequencing technology, researchers found that Tp53 mutations frequently occur in ENKTL. Ye Ziyin et al. showed that the high expression of tumor suppressor protein p53 encoded by the Tp53 gene was closely related to ENKTL staging (26). MicroRNA (miRNA) expression is another tumor-promoting pathway of chronic inflammation, which stimulates miRNA-155 overexpression and allows inflammatory cells to produce DNA-mutating mediators in a paracrine manner (24). Zhang Xudong et al. found that miRNA-155 was highly expressed in ENKTL cell lines SNK-6 and YTS, especially in advanced ENKTL patients (27, 28). These results suggest that CRS can promote T/NK cell aggregation and increase its genetic instability, which is a key driver of ENKTL at the genetic level. The difference between Tp53 mutation and miRNA-155 overexpression in ENKTL staging suggests that this may be one of the reasons for the correlation between the CRS and CA stages.

Second, in addition to genetic instability, abnormal proliferation of T/NK cells is another key process of ENKTL. CRS promotes proliferation and anti-apoptosis of the tumor. Long-term stimulation and release of a large number of inflammatory factors can activate nuclear factor-κB (NF-κB) and signal activator of transcription_3_ (STAT_3_). They have been involved in the NF-κB, and JAK-STAT signaling pathways play an important role as a bridge between tumor cells and inflammatory cells. Their activation causes the continuous release of cytokines and active mediators and induces malignancies. At the same time, they also enhance the proliferation and adhesion ability of malignant cells, promote the expression of anti-apoptotic genes, further release inflammatory mediators, and form a positive feedback amplification loop between inflammation and tumor (24). In addition, both NF-κB and STAT_3_ interfere with p53 synthesis and attenuate p53-mediated genomic surveillance (25). Abnormal activation of NF-κB and JAK-STAT_3_ signaling pathways have been demonstrated to be common in ENKTL (29–32). Therefore, we believed that there was also a positive feedback effect between CRS and ENKTL, and the amplification of the inflammatory effect not only promotes the occurrence of ENKTL but also promotes the transition of ENKTL from limited to the progressive stage. The accumulation of positive feedback effect may be another reason why the history length of CRS is correlated with ENKTL staging.

Third, the progression of ENKTL is a multistage process, and local invasion and distant metastasis of tumor cells are essential. A large number of inflammatory factors generated in the inflammatory microenvironment promote the establishment of TME, and tumor-associated macrophages (TAMs) are the most common immune cell in TME (33). TAMs also induced the production of vascular endothelial growth factor (VEGF) and matrix metalloproteinases (MMPs). They are necessary conditions for angiogenesis, tissue destruction, tumor invasion, and metastasis (34, 35). Wang et al. showed that TAMs content is closely related to clinical characteristics such as B symptom and LDH level of ENKTL patients, and high TAMs content may be a poor prognostic factor of ENKTL (36). We believe that long-term inflammatory stimulation increases the local TAMs content in the nasal cavity and promotes the expression of VEGF and MMPs, thus enhancing the invasion ability of ENKTL and promoting its invasion of surrounding tissues and distant metastasis, which may be an important reason why CRS affects PFS in ENKTL patients.

Fourth, the initiation and progression of ENKTL depend on the inhibition of normal immune response. The existence of tumor immune surveillance mechanism inhibits the occurrence and progress of tumor, but as a result of inflammatory factors of long-term stimulation and formation of TME, tumor cells constantly edit and adjust the host antitumor immune response, finally breaking the balance between the antitumor immunity and tumor-promoting immunity, resulting in immune escape (35, 37). The tumor-mediated immune regulation mainly affects the function of related immune cells by secreting soluble mediators. For example, the expression of programmed cell death-ligand 1 (PD-L1) can inhibit the killing ability of T cells (22). Wen et al. confirmed that the expression of PD-L1 is closely related to the progress of ENKTL, and it has been reported that PD-1/PD-L1 inhibitor can benefit ENKTL patients (38, 39). Therefore, inducing PD-L1 expression to help tumor cells escape immune surveillance may be one of the mechanisms by which CRS promotes ENKTL progress.

Finally, Epstein–Barr virus (EBV) has an infection rate of up to 90% in the population. After infection, EBV can lie dormant in B cells until it is reactivated and has a strong ability to promote lymphocyte growth and transformation, which is also considered an important pathogenic factor of ENKTL (40, 41). In our study, 60 (28.0%) of 214 ENKTL patients had elevated plasma EBV-DNA load, and EBV-encoded RNA (EBER) expression was positive in almost all patients. The presence of CRS causes immune dysfunction and may lead to the reactivation of EBV, which promotes the occurrence of ENKTL and promote its progression. Under the continuous stimulation of chronic inflammatory response or immune disorders, the infected cells enter the viral lysis cycle and transmit the virus to T cells or NK cells, leading to the occurrence of ENKTL (40, 41). EBV genomes can be embedded into host cells to produce various antigens, such as latent membrane protein (LMP1), LMP2A, and Epstein–Barr nuclear antigen1 (EBNA1) (42). LMP1 was considered as the most important oncoprotein of EBV-related proteins, which regulates NF-κB, Phosphatidylinositol-3-kinase (PI3K), JAK-STAT, and other signaling pathways and mediates proliferation, invasion, and metastasis of ENKTL tumor cells by upregulating expression of PDL1 (38, 39). LMP2A also induces epigenetic changes in the body genome through CpG island methylation and inactivates tumor suppressor genes such as PTEN and tumor-associated antigens (43). Therefore, we supposed that CRS and EBV are directly or indirectly related and promote each other to induce the occurrence and progress of ENKTL.

Based on the above discussion, we considered that the history of CRS was related to the CA stage of UAT-ENKTL patients, and the correlation may be related to the following factors. On the one hand, some studies have shown that the overall prevalence of CRS in the population was about 8%; among adults aged 15–75, it was 8.2%, which is much higher than the incidence of UAT-ENKTL (44). In addition, the early symptoms of ENKTL were not typical and did not attract enough attention, especially for ENKTL patients with a long history of CRS. The similar nasal symptoms and delayed clinical treatment result in the long history of CRS patients who had late staging after ENKTL onset. On the other hand, at the genetic level, the accumulation of Tp53 mutation and miRNA-155 overexpression; at the molecular level, accumulation of positive feedback effects between CRS and ENKTL caused by abnormal activation of molecular signaling pathways; and at the immune level, the degree of interference of the human immune system may be the reasons for the late CA staging in some UAT-ENKTL patients with a long history of CRS. The enhanced proliferation, anti-apoptosis, and invasion and metastasis of malignant T/NK cells stimulated by CRS may be the reasons why patients with a long history of CRS have poor PFS. In addition, EBV infection and autoimmune disorders may also play an important role. The disorder of the immune system plays a role in all stages of the occurrence, progression, invasion, and metastasis of ENKTL. The development and progression of ENKTL are determined by the game between anti- and pro-tumor immunity. The presence of CRS confuses the human immune environment and breaks the balance between anti- and pro-tumor immunity. ENKTL is the result of the interaction of many factors, among which the duration of CRS disease also plays an important role. More experimental and clinical evidence is needed to clarify the relationship between ENKTL and CRS in the future.

In this study, we also found that there were no significant differences in initial treatment outcomes between groups with different CRS histories, suggesting that CRS did not affect patients’ response to treatment, which may also be the reason why CRS did not affect patients’ OS. In addition, this may be related to individual differences and the potential limitations of this study. First, this study is a single centrality study with small sample size. Second, patients have certain subjectivity and memory bias when providing the history of CRS. Thus, it is necessary to conduct a large sample and multi-center study and add more comprehensive prognostic factors to further validate the association between CRS and ENKTL.

## Conclusion

In summary, CRS affects the occurrence and development of UAT-ENKTL through many links, and the infection of EBV and CRS also complements and promotes each other that has a direct or indirect impact on the occurrence and progress of UAT-ENKTL. Through this retrospective analysis, we found the correlation between the history of CRS and UAT-ENKTL patients’ staging and prognosis, which suggested that we should find and treat CRS as soon as possible in clinical practice, control chronic inflammation of the nasal cavity, and reduce the possibility of the occurrence or progression of UAT-ENKTL.

## Conflict of Interest

The authors declare that the research was conducted in the absence of any commercial or financial relationships that could be construed as a potential conflict of interest.

## Publisher’s Note

All claims expressed in this article are solely those of the authors and do not necessarily represent those of their affiliated organizations, or those of the publisher, the editors and the reviewers. Any product that may be evaluated in this article, or claim that may be made by its manufacturer, is not guaranteed or endorsed by the publisher.

## Data Availability Statement

The datasets used and analyzed during the current study are available from the corresponding author on reasonable request. Requests to access these datasets should be directed to mingzhi_zhang1@163.com.

## Ethics Statement

The studies involving human participants were reviewed and approved by the Ethics Committees of the First Affiliated Hospital of Zhengzhou University. Written informed consent to participate in this study was provided by the participants’ legal guardian/next of kin. Written informed consent was obtained from the individual(s), and minor(s)’ legal guardian/next of kin, for the publication of any potentially identifiable images or data included in this article.

## Author Contributions

MZ, LZ, and YC contributed to the design of the study. TL and YC collected the data. MZ provided methodological advice. TL and YC performed data and statistical analysis. TL wrote the first draft of the manuscript. MZ, LZ, and YC proofread the manuscript. MZ and LZ supervised the conduct of the study. All authors contributed to the article and approved the submitted version.

## Funding

This study was supported by the National Natural Science Foundation of China (Grant No. 81970184), the National Natural Science Foundation of China youth Science Foundation project (No. 82000204), and the Medical Science and Technology Research Project of Henan Province (No. 192102310116).
